# Toxicity Response and Swimming Speed Regularity in *Daphnia magna* After Short-Term Exposure to Diuron

**DOI:** 10.3390/toxics13050395

**Published:** 2025-05-15

**Authors:** Feihu Qin, Nanjing Zhao, Gaofang Yin, Yunfei Luo, Tingting Gan

**Affiliations:** 1 University of Science and Technology of China, Hefei 230026, China; qinfeihu@mail.ustc.edu.cn (F.Q.); yunfeiluo@mail.ustc.edu.cn (Y.L.); 2 Anhui Institute of Optics and Fine Mechanics, Hefei Institutes of Physical Science, Chinese Academy of Sciences, Hefei 230031, China; ttgan@aiofm.ac.cn; 3 Key Laboratory of Optical Monitoring Technology for Environment, Hefei 230031, China

**Keywords:** herbicide, DCMU, *Daphnia magna*, swimming behavior, toxic dose–effect

## Abstract

The agricultural production process contributes to the global issue of pesticide pollution. Based on the static toxicity test of diuron (DCMU) on *Daphnia magna* (*D. magna*) for EC_50_-48 h, a concentration range of 0.2 to 1 mg/L was set as sublethal concentrations, while lethal concentrations were set at 2 mg/L and 4 mg/L. This study analyzes the toxic response patterns of the swimming behavior indicators of *D. magna* exposed to different concentrations of DCMU. The results showed that the average speed (V) of *D. magna* decreased step by step with exposure time, regardless of exposure to sublethal concentration or lethal concentration. However, during the same short-term exposure period, the V of *D. magna* at lethal concentration was higher than that at sublethal concentration, which indicates that the swimming behavior of *D. magna* exposed to DCMU may be stimulated and accelerated. Compared to the control group, there is a statistically significant difference in the V of *D. magna* after short-term exposure, especially showing an extremely significant difference after 5 min of exposure. Evidently, compared to the traditional 48 h static toxicity testing method, the swimming behavior indicators of *D. magna* show a more sensitive response to DCMU after 5 min of exposure, making it more suitable for rapid toxicity detection. By expanding the range of exposure concentrations, it was found that the V indicator of *D. magna* responded significantly to a DCMU concentration of 0.05 mg/L after only 5 min of exposure, and a high degree of correlation was observed between the indicator and the exposure concentration. Through nonlinear fitting, the relationship between V and the dose–effect of DCMU toxicity presents an S-shaped curve, with R^2^ > 0.9. Consequently, it becomes possible to study the dose–effect relationship between the changes in the swimming behavior indicators of *D. magna* and the stress concentration based on this theory. This further establishes a foundation for the development of comprehensive aquatic toxicity rapid detection technology based on the toxic response of swimming behavior indicators.

## 1. Introduction

Despite the increasing number of studies indicating the ecological risks associated with the use of pesticides [1,2,3,4], the use of pesticides has still not achieved ecological sustainability [5,6,7]. While herbicides, as an important component of agricultural production activities, are widely used, they have already caused serious environmental pollution and the destruction of aquatic ecosystems [5,8,9]. Herbicides usually have high chemical stability and environmental persistence and generally have a low utilization rate in the application process [10,11,12,13], resulting in most of the chemicals and their derivatives entering the air, water, soil and other environmental media [2,4,14] and eventually entering the water through atmospheric transmission, surface runoff and other ways and causing water ecological environment pollution. Long-term accumulation may lead to the accumulation of pesticides in non-target organisms in the water environment, which has negative effects [15,16]. Aquatic organisms, whether exposed to pesticide-contaminated water bodies in the long or short term, may have their growth and reproductive capabilities affected. Moreover, such impacts may enter the food chain through bioaccumulation or biomagnification, causing long-term effects on higher organisms [10,17]. Reports from various places all show that herbicides are one of the most commonly detected pollutants in the environment, especially in watersheds near agricultural lands, where the pollution problem of multiple herbicides is particularly prominent [1,2,14,18]. Therefore, herbicides that remain in the environment can not only cause irreversible ecological damage but also pose health risks, which have always been the focus of water ecological risk research.

Diuron (DCMU) is a broad-spectrum phenylurea herbicide, which has been widely used in agriculture due to its effective control of a variety of field weeds, especially playing a key role in the management of crop seedlings [9,19]. However, in recent years, DCMU has been frequently found in freshwater and marine environments [20,21,22,23,24,25], posing a potential threat to the environment and human health. Research indicates that DCMU has significant toxic effects on aquatic organisms, posing serious ecological risks to non-target biological communities [26,27,28]. In particular, biological groups that are physiologically similar to the target species, such as phytoplankton, can have their photosynthesis inhibited in water bodies by DCMU, which hinders their growth and reproduction, causing them to exhibit extremely high biological toxicity [23,29,30]. In addition, the transfer of DCMU along the food chain can accumulate in consumer-level organisms, thereby having negative impacts on higher organisms. Experiments have shown that phenylurea herbicides can induce blood toxic effects in rats [31], and Wistar rats treated with DCMU experience a series of systemic and organ-specific toxic reactions, which may include effects on the immune system, blood system and other organs [32]. Similarly, DCMU has also been proven to induce effects on the thyroid function of fish, leading to a dynamic imbalance of the thyroid hormones thyroxine (T4) and triiodothyronine (T3) in the body, thereby causing adverse reactions in growth and basal metabolism [33,34]. Marine fish exposed to DCMU environments for long periods may experience chronic toxic effects (including hormone levels, immune function, and liver function), affecting the reproduction, immunity, and health of the fish [26]. In addition, some studies have shown that DCMU also has a certain chronic toxic effect on *cladocerans*, among which the lowest observable effect concentration on reproduction inhibition of *Daphnia pulex* for 7 days is 7.7 mg/L [35]. Although physiological indicators of lethal or reproductive effects are considered effective indicators for toxicity assessment [36], such toxicity evaluation methods have a long cycle and lack an understanding of adverse reactions before death. In general, the concentrations of most pollutants in the environment are not high enough to cause direct lethal damage to aquatic organisms [37], but they may still have adverse effects on the growth, development, reproduction, behavior, and physiological functions of organisms. Therefore, we need to pay more attention to the sublethal toxic effects that pollutants may cause before they cause biological death. However, there are few reports on the potential sublethal impacts of DCMU on aquatic organisms after short-term exposure.

*Daphnia magna* is a widely distributed cladoceran aquatic organism in freshwater ecosystems and is also one of the commonly used model organisms in toxicological research [38]. *D. magna*, as a primary consumer in the food chain, plays a pivotal role in connecting different trophic levels and is an important component of the ecosystem [38,39,40]. Studies indicate that *D. magna* shows high sensitivity to various environmental stressors in aquatic ecosystems, including conventional pollutants such as pesticides [41], heavy metals [42], pharmaceuticals [43], and microplastics [44], making it an ideal experimental subject for aquatic toxicity studies. Traditional toxicological research relies on acute toxicity tests (OECD 202) or chronic reproductive toxicity tests (OECD 229) using *D. magna* to assess the toxicity of chemicals, but these methods may not cover the sensitive detection of sublethal toxic effects. Research has found that behavioral disturbances almost always occur before lethal effects, and the concentrations causing behavioral disturbances are significantly lower than those causing lethality [45]. Compared to acute lethality or other biomarkers (such as reproduction and growth), sublethal swimming behavior indicators show more sensitive stress responses [46]. Bownik reviewed the study of swimming behavior of *D. magna* as a biomarker in toxicity assessment, and the analysis of its swimming behavior is considered a new method for toxicity evaluation and water quality monitoring, further indicating that sublethal locomotor behavior can serve as a toxicity endpoint [47].

The swimming behavior of animals is influenced by the integrated effects of their physiological, sensory, nervous, and muscular systems. The use of video-based animal behavior tracking system to analyze digital video files has become a standard means to obtain biological behavioral data in ecotoxicology and behavioral studies [48]. By measuring the locomotor parameters of *D. magna*, such as speed, acceleration, and distance traveled, one can describe their locomotor behavior, which can reflect the impact of chemicals on sensitive systems such as the nervous and endocrine systems. Previous research has indicated that the swimming behavior of *D. magna* exhibits rapid responses to mercury chloride toxicity. Notably, V is more suitable as a sensitive indicator for rapid detection of toxic stress responses compared to other parameters such as the distance traveled or acceleration [49]. Although the locomotor behavior of *D. magna* is considered a reliable biomarker for assessing the adverse effects of environmental pollutants, previous studies have often only demonstrated significant impacts of multiple pollutants on their locomotor behavior [41,42,43,44]. Fewer studies have utilized these sensitive locomotor indicators to further evaluate the dose–effect relationships of pollutants. This limits quantitative analysis and decision making regarding sublethal toxic effects. Therefore, this study employs *D. magna* as a model organism to investigate behavioral parameter variations under different DCMU exposure concentrations. By establishing quantitative relationships between toxic stress and behavioral responses, we aim to validate the feasibility of a rapid toxicity assessment method based on *D. magna* locomotion, thereby providing scientific support for comprehensive aquatic toxicity evaluation.

## 2. Materials and Methods

### 2.1. Materials

The chemical DCMU used in this experiment comes from Aladdin (Shanghai, China) Reagent Company. According to the physicochemical characteristics of DCMU, its solubility in water at 25 °C is 42 mg/L, and the lethal concentration LC50 for aquatic invertebrates exposed to DCMU for 48 h is distributed within the range of 1–2.5 mg/L [50]. In accordance with the OECD 202 guidelines, if *D. magna* sinks to the bottom of the multi-well test plate and shows no swimming behavior within 15 s, it is considered to be immobile. This criterion is used as the endpoint for assessing toxicity in static toxicity tests, aiming to determine the effective concentration of DCMU that causes 50% of *D. magna* to lose their mobility after 48 h of exposure. According to the ecological toxicity information in the official Material Safety Data Sheet (MSDS) for DCMU, the static toxicity test EC_50_-48 h for *D. magna* is 1.4 mg/L. Therefore, the exposure experimental concentration range set in this study was divided into sublethal and lethal. The sublethal concentration was set to be less than 1 mg/L, specifically 0.2 mg/L, 0.4 mg/L, 0.6 mg/L, 0.8 mg/L and 1 mg/L. Lethal concentrations were set at 2 mg/L and 4 mg/L.

### 2.2. Domestication of Organisms

The model organism selected in this study was female *D. magna* (<24 h), which was procured from the Guangdong Laboratory Animal Monitoring Institute (Guangdong, China). *D. magna* were placed in a constant-temperature light incubator (temperature was maintained at 22 ± 1 °C, the light intensity set at 2000 lux, and a light–dark cycle maintained for 16 h:8 h) for expanded breeding. They were regularly fed with *Chlorella* sp. (concentration approximately 10^5^ cells/mL) as feed every day. The culture medium used was filtered tap water that had been aerated for over 48 h (pH 7.0–8.5, dissolved oxygen above 4 mg/L, hardness 250 ± 22 mg/L). The tap water was filtered using a water purifier (J1105-ROB8, Shenzhen, China). During the long-term domestication culture, the culture medium was replaced every three days by siphon to ensure that there was no obvious metabolite precipitation at the bottom of the culture vessel. In the exposure experiments, the juveniles used were all derived from purebred female *D. magna* that had been continuously cultured in the laboratory environment for at least one month. These were obtained through parthenogenetic reproduction.

### 2.3. Exposure Experiment

The juveniles (<24 h) were obtained by performing two rounds of siphoning and sieve filtering on the culture medium of *D. magna* that had been continuously cultured for over one month. The specific operating procedure can be referred to in previous related studies [49]. The exposure experiment was divided into the control group and experimental group. Before the start of the toxicity exposure experiment, 96 individuals (<24 h) were randomly prepared and divided into 7 experimental groups, with 12 juveniles per group, and the remaining individuals served as the control group. The individuals were placed in circular grooves (D = 25 mm, H = 17 mm) of a multi-well plexiglass reaction plate, with one individual per recess, and the culture medium was injected for a 5 min environmental adaptation. The control group of *D. magna* was exposed to the culture medium, while the experimental groups of *D. magna* were exposed to different gradient concentrations of DCMU solution. First, the culture medium in the circular recesses was replaced with the corresponding concentration of DCMU solution. The groove moistening pretreatment was carried out before the replacement, and then the moistening solution was sucked out and added to the DCMU solution again to avoid affecting the measurement results. After exposure to DCMU solution, each group was continuously followed for 35 min.

### 2.4. Swimming Behavior Collection

The swimming behavior of *D. magna* was recorded in real time using a camera and saved. By adjusting the angle and focus of the camera, the swimming trajectory of *D. magna* was ensured to be within the field of view of the lens, and continuous shooting was performed at 30 fps for 35 min to obtain the complete swimming behavior dataset of *D. magna* during short-term exposure. Subsequently, video frames were extracted from the recorded video to obtain the image data of *D. magna*. By annotating the swimming images of *D. magna*, a training dataset for the target recognition of *D. magna* is obtained. Then, based on deep learning, the original video training dataset is trained to construct a data model for the target detection of *D. magna*. Subsequently, the trained data model is loaded using the YOLOX [51] object detection algorithm to perform target recognition on each frame of the original video, which serves as the detector for the Multi-Object Tracking (MOT) algorithm. Finally, the ByteTrack [52] algorithm is combined to perform the frame-by-frame recognition and tracking of each video segment, outputting the coordinate sequences of the continuous movement of *D. magna*.

**Figure 1:**
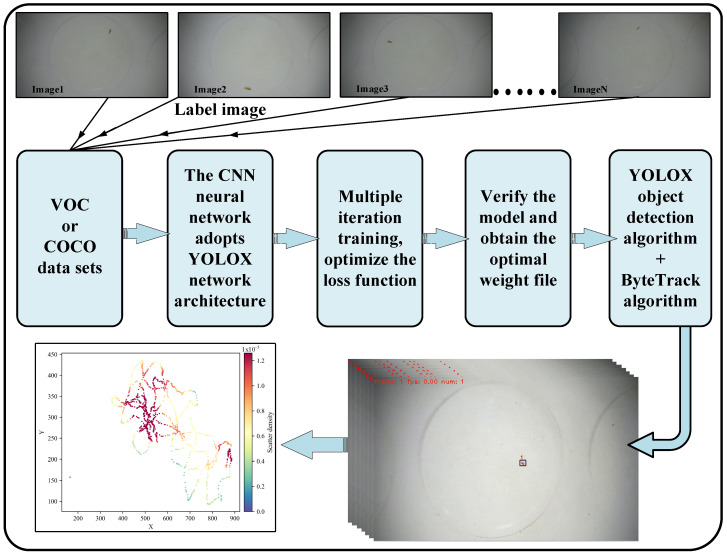
Flowchart of collection of swimming behavior indicators of *D. magna*.

During the process of target tracking and detection, the continuous swimming path of *D. magna* is recorded by monitoring the coordinates of the top-left corner of its bounding box. In chronological order, the coordinates of the target’s bounding box in the first frame of the video sequence are recorded as (x_0_, y_0_). Subsequently, the coordinates of the target’s bounding box in each subsequent frame are recorded as (x_1_, y_1_), and so on, until (x_i−1_, y_i−1_) in the i-th frame. The Euclidean Distance formula is used to calculate the distance between adjacent points in space, thereby obtaining the total distance traveled by the target during the tracking period, which is the cumulative distance (L) indicator within the continuous tracking cycle. The frame rate of the video is used to determine the time difference between consecutive frames, and then the speed calculation formula is applied to obtain the swimming speed of *D. magna* between these two frames. In order to calculate the average speed of *D. magna* over the entire tracking process, the L indicator values of *D. magna* between consecutive frames are accumulated, and the total displacement is divided by the total time span to obtain the result. See Figure 1.

Additionally, to reduce the impact of frame loss during the target recognition process on the statistical results of indicators, this study primarily focuses on the V indicator of the swimming behavior of *D. magna*. By statistically measuring the V indicator of *D. magna* every 5 min, the changes in this swimming behavior indicator over a 35 min period are continuously observed.

### 2.5. Data Processing

In this study, Python 3.7 was used for data analysis to investigate the effect of DCMU on the average speed index of *D. magna*. The swimming behavior parameters were measured at different DCMU concentrations, and the trend chart of indexes was drawn with Origin software 2025 (10.2). A statistical analysis of the data was performed using IBM SPSS Statistics 19 software, and *p* < 0.05 was taken as the standard of statistical significance, and the average value was taken. To assess the differences between the experimental and control groups, a one-way analysis of variance (ANOVA) and Tukey’s post-hoc multiple comparison test were used, the Kolmogorov–Smirnov test was used to assess the normal distribution of the data, and the homogeneity of the variance of the data was tested by the Levene test.

## 3. Results and Discussion

### 3.1. Changes in the Average Speed V Index Exposed to DCMU

**Figure 2:**
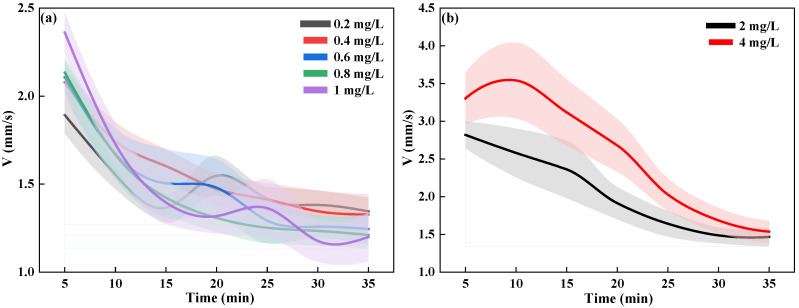
Dynamic changes in the speed (V) indicators of *D. magna* continuously exposed to DCMU for 35 min. (**a**) The dynamic changes in the V indicators for *D. magna* exposed to sublethal concentration ranges; (**b**) the dynamic changes in the V indicators for *D. magna* exposed to lethal concentrations. The results are expressed as mean ± SE. The error bars, centered around the mean, are represented by shaded areas in the graph, with different colors indicating different exposure concentrations.

As shown in Figure 2, the results of the continuous tracking of the swimming behavior of *D. magna* showed that when exposed to 0.2–1 mg/L sublethal DCMU solution for 35 min, the average speed V index of *D. magna* decreased from about 2.5 mm/s to less than 1.5 mm/s. Relative to the sublethal exposure conditions, the V index of *D. magna* exposed to 2 mg/L and 4 mg/L DCMU solutions for 35 min decreased from about 3.5 mm/s to about 1.5 mm/s. In addition, under the same exposure time conditions, the V of *D. magna* at the lethal concentration is always higher than that at the sublethal concentration, especially in the first 15 min of exposure, where the difference is more pronounced.

Whether exposed to sublethal or lethal concentrations, the overall trend of the V indicator of *D. magna* with exposure time is consistent. In both cases, the V indicator decreases gradually from a high level as the exposure time increases. The difference lies in the fact that the V indicator of *D. magna* exposed to lethal concentrations is higher than that of those exposed to sublethal concentrations, indicating that the swimming behavior of *D. magna* exposed to DCMU in the short term is subject to a specific stress response.

Research has demonstrated that DCMU and its metabolites exhibit multi-level toxic effects on aquatic organisms. In terms of oxidative stress, studies using the *Crassostrea gigas* embryo–larval bioassay have identified the excessive accumulation of reactive oxygen species (ROS) as a key contributing factor [53]. This oxidative stress can induce cellular damage including lipid peroxidation, protein denaturation, and DNA impairment, potentially leading to apoptosis or necrosis [53]. Considering that both *D. magna* and oysters are aquatic invertebrates, DCMU may disrupt the redox balance in *D. magna* through similar mechanisms (e.g., by inducing ROS accumulation), which could account for the abnormal swimming speed observed in short-term exposure experiments.

Notably, DCMU’s toxicity mechanisms extend beyond oxidative stress. In vertebrates, substantial evidence demonstrates that DCMU disrupts the endocrine system, affecting growth, development, and reproduction [26,33,34]. For invertebrates like *D. magna*, direct evidence of endocrine disruption remains limited; however, the evolutionary conservation of ecdysteroid signaling pathways (known to regulate molting and reproduction in arthropods) suggests potential vulnerability. Chronic toxicity to cladocerans has been confirmed, with reproductive inhibition as a sensitive endpoint [35]. Under prolonged exposure, oxidative stress may interact with endocrine modulation to exacerbate ecological risks.

### 3.2. Significance Analysis of V Exposed to DCMU

According to the design of the DCMU toxicity exposure experiment, during the continuous exposure period, the V indicator of *D. magna* was recorded every 5 min within each interval, with the continuous tracking of the changes in the speed of *D. magna*.

**Figure 3:**
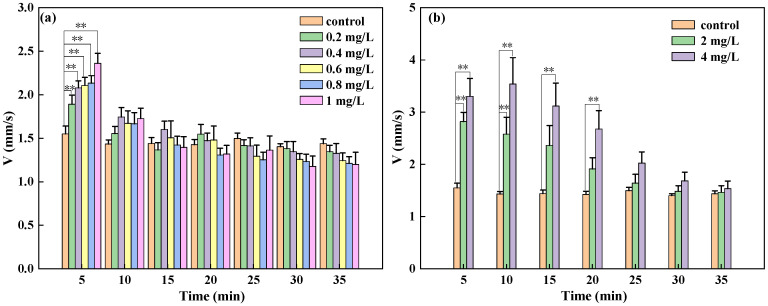
The one-way ANOVA of the average speed of *D. magna* in different DCMU concentrations compared to the control group. (**a**) The significance of the sublethal concentration range exposure for *D. magna*; (**b**) the significance of the lethal concentration range exposure for *D. magna*. The results are expressed as mean ± SE. Two asterisks (**) indicate highly statistically significant results (*p* < 0.01).

The results shown in Figure 3 indicate that during the 5 min exposure to sublethal doses of DCMU, the V of *D. magna* significantly increased, with a highly significant difference compared to the control group. After continuous exposure for 10 min, the V of *D. magna* in the treatment group was overall higher than that in the control group. However, compared to the V of *D. magna* in the first 5 min mark, there was a downward trend. Moreover, the speed of *D. magna* in the concentration range of 0.2 to 0.1 mg/L showed no significant difference to that of the control group. It was not until continuous exposure for 30 min that the swimming speed of *D. magna* in the concentration range of 0.2 to 1 mg/L became lower than that of the control group. Similarly, after short-term exposure to lethal doses of DCMU, the V of *D. magna* significantly increased, then gradually decreased with exposure time, until after 30 min the swimming speed of *D. magna* essentially returned to the level of the control group. Therefore, the swimming behavior of *D. magna* exposed to DCMU for a short period undergoes significant changes, especially in the first 10 min, where the impact is highly significant.

According to the Step Stress Model (SSM) proposed by Gerhardt et al., it is assumed that organisms, when exposed to pollutants exceeding their respective resistance thresholds, will exhibit a time-dependent sequence of stress responses involving different regulatory or compensatory behaviors [54]. Therefore, when faced with continuous or gradually increasing stressors or stress sources, organisms activate and carry out a series of physiological adjustments and behavioral stress responses to adapt to these external pressures. When exposed to pollution pulses, the first response may be avoidance (increased swimming), and this gradual stress phenomenon has been observed in a variety of aquatic organisms [54,55,56]. According to our exposure experiment results, as the exposure time increases, the average speed V of *D. magna* also undergoes a stepwise regulatory change process. Compared to previous studies on the swimming behavior of *D. magna* under the stress of acidic mine drainage following the SSM [54], it further indicates that there are gradual stress characteristics when *D. magna* is exposed to DCMU in the short term.

The experimental results demonstrated that, whether exposed to sublethal or lethal concentrations, *D. magna* exposed to DCMU for a short period will be stimulated to move faster, and a significant stress response is observed within 5 min of exposure, indicating that behavioral parameters can reflect sublethal toxic responses. Compared to the 48 h static toxicity test EC_50_ indicator, the toxic response of swimming behavior is more sensitive, and the lower limit of the response concentration is lower, indicating that behavioral responses may be faster and more sensitive toxicological parameters than mortality [45,46]. However, further validation would be required to determine whether this sensitivity advantage applies to other classes of pesticides with different modes of action.

### 3.3. Relationship Between Swimming Behavior and Dose–Response

**Figure 4:**
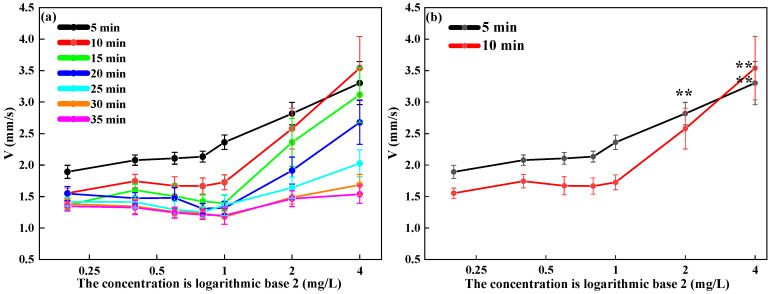
The change in the V of *D. magna* with exposure concentration under the same exposure time. (**a**) The change in the V index of *D. magna* with exposure concentration; (**b**) the statistical differences in the V index of *D. magna* with exposure concentration at 5 min and 10 min exposures. The exposure concentration on the x-axis is presented in logarithmic form, and the results are expressed as mean ± SE. Two asterisks (**) indicate results that are highly statistically significant (*p* < 0.01).

This paper further verifies the functional relationship between the swimming behavior indicators of *D. magna* and the toxic dose–response effect by expanding the range of exposure concentrations, thereby obtaining the lower limit of DCMU toxicity response. As shown in Figure 4, the changes in the swimming behavior indicators of *D. magna* during the 35 min short-term continuous exposure period with varying exposure concentrations can be seen. In the first 10 min of exposure, the V indicator of *D. magna* shows an increasing trend with the increase in exposure concentration, especially in the lethal concentration range. However, after 10 min of exposure, the V indicator of *D. magna* only shows an increasing trend in the lethal concentration range.

The results indicate that, within the same exposure time, the V indicator of *D. magna* correspondingly increases with the increase in exposure concentration, especially in the first 10 min of exposure, where this phenomenon is particularly pronounced. Further observations revealed that, at the same sublethal concentration, the change in V of *D. magna* is essentially not significant. However, under lethal concentrations, there is a significant difference in the change in V. Biological toxic effects are typically influenced by two main factors: exposure time and exposure concentration. These two factors interact with each other to jointly determine the toxic response of organisms [57]. Therefore, the experimental results reveal that at low concentrations, the changes in the swimming behavior of *D. magna* are primarily regulated by exposure time, while at high concentrations, the changes in swimming behavior are mainly dominated by the exposure dose. This result is consistent with Haber’s Law of toxic dose–effect, which states that time and dose have different impacts on biological behavior, and these factors together influence the overall response of organisms to toxic stress.

**Figure 5:**
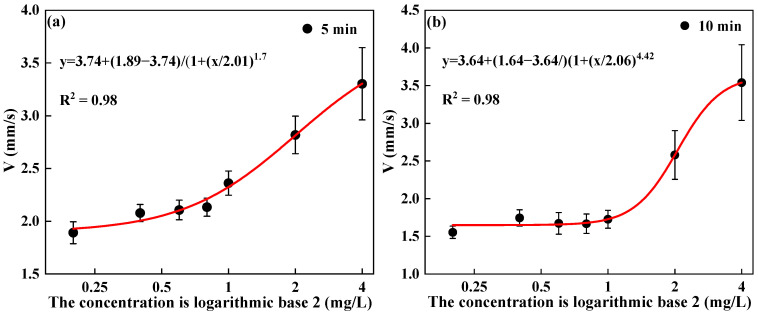
The functional fitting of the V of *D. magna* with exposure concentration. (**a**) The nonlinear fitting of the V of *D. magna* exposed for 5 min with the exposure concentration; (**b**) the nonlinear fitting of the V of *D. magna* exposed for 10 min with the exposure concentration. The exposure concentration on the x-axis is presented in logarithmic form, and the results are expressed as mean ± SE, with R^2^ > 0.9.

This study establishes a mathematical model describing the relationship between the toxic effects and exposure dose of *D. magna* after exposure to DCMU. To this end, this study specifically analyzed the correlation between the swimming speed of *D. magna* and the exposure concentration at two different exposure intervals of 5 min and 10 min. As shown in Figure 5, through nonlinear fitting, it was found that there is a very good S-shaped curve relationship between the V indicator of *D. magna* and the exposure dose, with R^2^ > 0.9. This phenomenon is consistent with the general pattern of toxic stress. Therefore, based on the mathematical model of the relationship between the characteristic indicators of *D. magna*’s swimming behavior and the dose–effect, a quantitative analysis of toxicity can be achieved.

**Figure 6:**
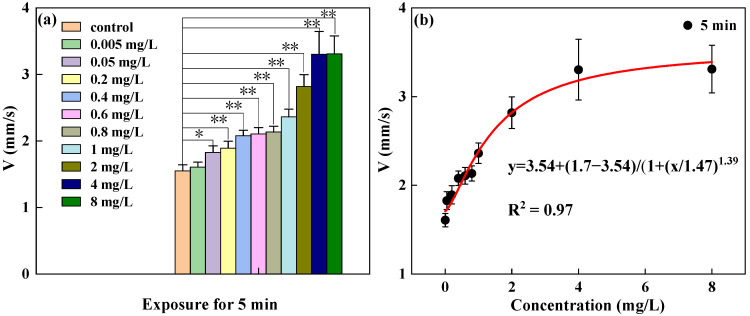
The toxic response and variation pattern of the V index of *D. magna* exposed for 5 min. (**a**) The statistical differences in the V of *D. magna* at different exposure concentrations relative to the control group; (**b**) the nonlinear fitting between the V of *D. magna* and the exposure concentration. The results are expressed as mean ± SE, with the fitting determination coefficient R^2^ > 0.9. An asterisk (*) indicates results that are statistically significant (*p* < 0.05); two asterisks (**) indicate results that are highly statistically significant (*p* < 0.01).

**Figure 7:**
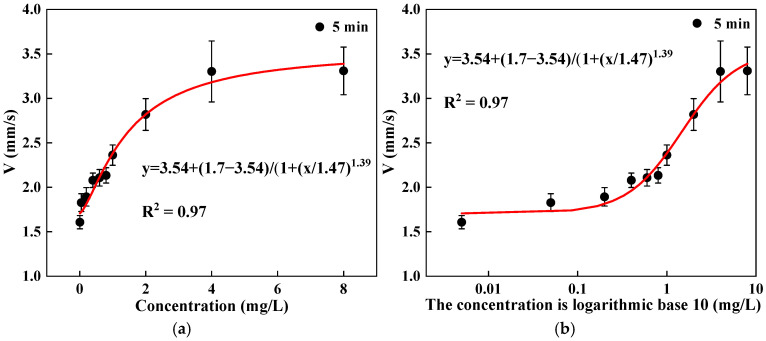
The nonlinear fitting of the V index of *D. magna* with exposure concentration after a short-term exposure of 5 min. (**a**) The nonlinear fitting of the V of *D. magna* with the exposure concentration; (**b**) the nonlinear fitting between the relative change in the V index of *D. magna* and exposure concentration. The exposure concentration on the x-axis is presented in logarithmic form, and the results are expressed as mean ± SE, with the fitting determination coefficient R^2^ > 0.9.

Based on a 5 min short-term exposure, changes in swimming behavior indicators were observed under concentration conditions of 0.005, 0.05, and 8 mg/L. As shown in Figure 6, after 5 min of exposure, the V indicator of *D. magna* showed a statistically significant difference compared to the control group, with significant changes in the V indicator of *D. magna* in the exposure range of 0.05 to 8 mg/L. However, there was no statistically significant difference under the exposure concentration of 0.005 mg/L, indicating that based on a short-term 5 min exposure, the swimming indicator V of *D. magna* can respond to the toxic effect of DCMU at a concentration of 0.05 mg/L. By performing a nonlinear fit of the changes in exposure concentration and the V indicator of *D. magna*, the results satisfied an S-shaped curve relationship. The results further indicate that the changes in the swimming indicators of *D. magna* exposed to DCMU exhibit an S-shaped curve stress effect relationship. Therefore, based on this mathematical model, further research can be conducted on the dose–effect relationship between the changes in the swimming behavior of *D. magna* and the stress concentration. See Figure 7.

To further reflect the impact of DCMU on the V index of *D. magna*, this paper uses the relative change in the V index of *D. magna* for a quantitative description. Based on the 5 min short-term exposure, the change in the V index of *D. magna* shows a good correlation with the exposure concentration, still conforming to an S-shaped curve relationship.

## 4. Conclusions

Herbicides, as a widely used class of pesticides, pose ecological safety risks to aquatic environments. Since DCMU can induce the metabolism and endocrine system of aquatic organisms, it directly or indirectly affects the normal physiological activities of organisms. If this continues over time, it will seriously harm the health and survival of aquatic organisms and may even affect higher organisms. To better and more quickly understand the impact of DCMU on organisms, the results of this study indicate that the swimming behavior of *D. magna* exposed to DCMU is disrupted to varying degrees. Compared to the traditional 48 h stress test, this study found that under a 5 min stress period, the sublethal swimming behavior of *D. magna* showed a significant response relative to the control group’s swimming patterns. This suggests that the swimming behavior indicators of *D. magna* can serve as sensitive biomarkers for the toxicity assessment of DCMU and are more suitable for rapid screening in comprehensive aquatic toxicity testing. Subsequent studies further confirmed that there is a good correlation between the swimming behavior indicators of *D. magna* and the exposure concentration, with the relationship manifesting as an S-shaped inhibition curve, which highly coincides with the stress effects produced by toxic substances on model organisms. Therefore, the S-shaped curve can reveal the association between the concentration of stressors and toxic effects, providing a theoretical basis for studying the dose–effect relationship between changes in the swimming behavior of *D. magna* and stress concentration. While the generalizability of this method to other pesticides remains to be tested, this approach establishes a framework for future investigations into behavior-based contaminant screening.

## Data Availability

The data that support the findings of this study are available from the corresponding author upon reasonable request.
